# The gut microbiota in young and middle-aged rats showed different responses to chicken protein in their diet

**DOI:** 10.1186/s12866-016-0895-0

**Published:** 2016-11-25

**Authors:** Yingying Zhu, He Li, Xinglian Xu, Chunbao Li, Guanghong Zhou

**Affiliations:** 1 Key Laboratory of Meat Processing and Quality Control, MOE, Key Laboratory of Animal Products Processing, MOA, Jiang Synergetic Innovation Center of Meat Processing and Quality Control, Synergetic Innovation Center of Food Safety and Nutrition, Nanjing Agricultural University, No. 1 Weigang Road, Nanjing, Jiangsu Province 210095 People’s Republic of China; 2 School of Biological and Medical Engineering, Hefei University of Technology, Hefei, 230009 China

**Keywords:** Gut microbiota, Aging, Chicken protein, Old rats

## Abstract

**Background:**

Meat protein in the diet has been shown to be beneficial for the growth of *Lactobacillus* in the caecum of growing rats; however, it is unknown whether gut microbiota in middle-aged animals have the same responses to meat protein diets. This study compared the composition of the gut microbiota between young and middle-aged rats after being fed 17.7% chicken protein diet for 14 days.

**Methods:**

Feces were collected on day 0 and day 14 from young rats (4 weeks old) and middle-aged rats (64 weeks old) fed with 17.7% chicken protein diets. The composition of the gut bacteria was analyzed by sequencing the V4-V5 region of the 16S ribosomal RNA gene.

**Results:**

The results showed that the composition of the gut microbiota was significantly different between young and middle-aged rats on both day 0 and day 14. The percentage of *Firmicutes* decreased for middle-aged rats (72.1% versus 58.1% for day 0 and day 14, respectively) but increased for young rats (41.5 versus 57.7% for day 0 and day 14, respectively). The percentage of *Bacteroidetes* increased to 31.2% (20.5% on day 0) for middle-aged rats and decreased to 29.6% (41.3% on day 0) for young rats. The relative abundance of the beneficial genus *Lactobacillus* increased in response to the intake of chicken protein in the young group, while it had the opposite effect in the middle-aged group.

**Conclusion:**

The results of our study demonstrated that 17.7% chicken protein diet promoted the beneficial genus *Lactobacillus* in young rats, but the opposite effect were found in the middle-aged group. To evaluate the linkage between diet and host health, age effect should be considered in the future studies.

**Electronic supplementary material:**

The online version of this article (doi:10.1186/s12866-016-0895-0) contains supplementary material, which is available to authorized users.

## Background

Gut bacteria have been shown to play a critical role in multiple physiological changes of the host related to metabolic disorders, immunity, brain development and many other aspects [1]. A lot of factors, including the diet and the age of host, can affect the composition of gut bacteria and subsequently host health.

In human and model animal studies, aging-related physiological changes in the gastrointestinal tract, e.g., inflammation and immunosenescence have been shown associated with the composition of gut microbiota [2–5]. Immunosenescence, a dysregulation of the immune system with age, may be related to antigen load from gut microbiota and affect the homeostatic balance of the gut microbiota [6]. In many cases, meat dish is one of the main courses for consumers over a large age span from the young to the adult and even to the elderly because meat is believed to be a good source of high-quality protein and other nutrients [7]. However, epidemiological studies have shown that human bodies may give different responses to meat intake with age [8, 9], and its underlying mechanism needs further study. Human nutrition studies are sometimes difficult to realize because of complex individual variations in background gut bacteria, behavior control and high cost, and model animal studies are the good choices and have been widely used. In a model animal study, we found that the intake of meat protein, especially chicken protein, may promote the growth of the beneficial genus *Lactobacillus* and maintain a more balanced composition of gut bacteria in growing rats as compared to soy protein diet [10]. However, it is still less known whether intake of chicken protein has the same impact on gut bacteria of middle-aged rats. The present study compared the composition of fecal microbiota between young and middle-aged rats after being fed chicken protein diet, and investigated whether intake of chicken protein (17.7% in diet) had the similar effectiveness to promote growth of beneficial gut bacteria in middle-aged rats to that in growing rats.

## Methods

### Animals and diets

A total of 15 male Sprague-Dawley rats were used, including eight growing rats (4 weeks old) from the Zhejiang Experimental Animal Center (Zhejiang, China, SCXK9<Zhejiang>2008-00), and seven middle age rats (64 weeks old) from the Academy of Military Medical Sciences Experimental Animal Center (Beijing, China, SCXK<Jun>2012-0004). The animal age phases were evaluated according to Flurkey, Currer and Harrison (2007) [11]. These two centers have the similar facility and quality control systems approved by the Bureau of Quality and Technical Supervision, P.R. China and the rats from the centers have the same backgrounds. In this case, the differences between the two centers were minimized. The rats were reared in a specific pathogen-free (SPF) animal center (SYXK<Jiangsu>2011-0037). The Ethical Committee of the Experimental Animal Center of Nanjing Agricultural University approved the experimental protocol. All rats were acclimatized for 7 days (fed with 17.7% casein chow diet) with a new environment that had a 12 h light-dark cycle and constant temperature and humidity (20.0 ± 0.5 °C, 60 ± 10%). After acclimatization, all rats were fed the formulated chicken protein diet. The animals were individually housed in plastic cages and given water and diet ad libitum.

Animal diets were formulated according to the recommendation of the American Institute of Nutrition (AIN-93G) [12]. The diets were composed of chicken protein powder (19.2%), cornstarch (39.75%), dextrinized cornstarch (13.2%), sucrose (10%), soybean oil (7%), fiber (5%), mineral mix (3.5%), vitamin mix (1%), choline bitartrate (0.25%), tert-butylhydroquinone (0.0014%) and moisture (18.2%). Protein powder was extracted from chicken *pectoralis major* muscle as previously described [10], and the protein percent was 92.4%.

### Sample collection

Feces were collected from growing and middle-aged rats on day 0 and day 14. All fecal samples were immediately frozen in liquid nitrogen and then transferred to −80 °C for analyses of gut bacteria composition.

### Bacterial community analyses and Bioinformatics

DNA was extracted using the QIAamp DNA Stool Mini Kit (NO.51504, Qiagen, Germany) according to the manufacturer’s protocol. The V4-V5 region of the 16S rRNA gene was selected for amplification from DNA samples. The universal primers used were F515 (5′-GTGCCAGCMGCCGCGG-3′) and R907 (5′-CCGTCAATTCMTTTRAGTTT-3′) which also carried an eight-base unique sequence (so-called barcode) for each sample. PCR reactions were run and amplicons were sequenced as previously described [10].

Bioinformatics analysis was referred to our previous study [10]. Raw fastq files were demultiplexed and quality-filtered using QIIME (version 1.17). Briefly, the 250 bp reads were truncated at any site receiving an average quality score <20 over a 10 bp sliding window. The truncated reads shorter than 50 bp were removed. Exact barcode matching was defined with not more than 2 bp mismatching with primer. Reads containing ambiguous characters were removed. The sequences that overlap longer than 10 bp were assembled according to their overlap sequence. Reads that could not be assembled were discarded. Operational Taxonomic Units (OTUs) were clustered with 97% similarity cutoff using UPARSE (version 7.1 http://drive5.com/uparse/) and chimeric sequences were identified and removed using UCHIME. The phylogenetic affiliation of each 16S rRNA gene sequence was analyzed by RDP Classifier (http://rdp.cme.msu.edu/) against the silva (SSU119) 16S rRNA database using confidence threshold of 70%. Rarefaction analysis and alpha diversity were performed using Mothur. Community diversity was evaluated by Shannon index and Simpson index. Community richness was evaluated by Chao and ACE. The heatmap was preformed by the R package (R 3.0.2).

### Statistical analysis

Differences in the relative abundance of fecal microbiota on the phylum and genus levels between two groups at two time points were evaluated by factorial analysis of variance (ANOVA). Means were compared by Duncan’s multiple comparison under the SAS system (version 9.2). Significance was set below 0.05 for all statistical analyses.

LEfSe analysis was performed (http://huttenhower.sph.harvard.edu/galaxy/) to find the the differences between two or more biological conditions (or classes) [13]. The differences in features were identified at the OTU level. The LEfSe analysis conditions were as follows: 1) alpha value for the factorial Kruskal-Wallis test among classes was less than 0.05; 2) alpha value for the pairwise Wilcoxon test among subclasses was less than 0.05; 3) the threshold on the logarithmic LDA score for discriminative features was less than 2.0; 4) multi-class analysis was set as all-against-all.

## Results

### Richness and diversity of gut microbiota

**Fig. 1:**
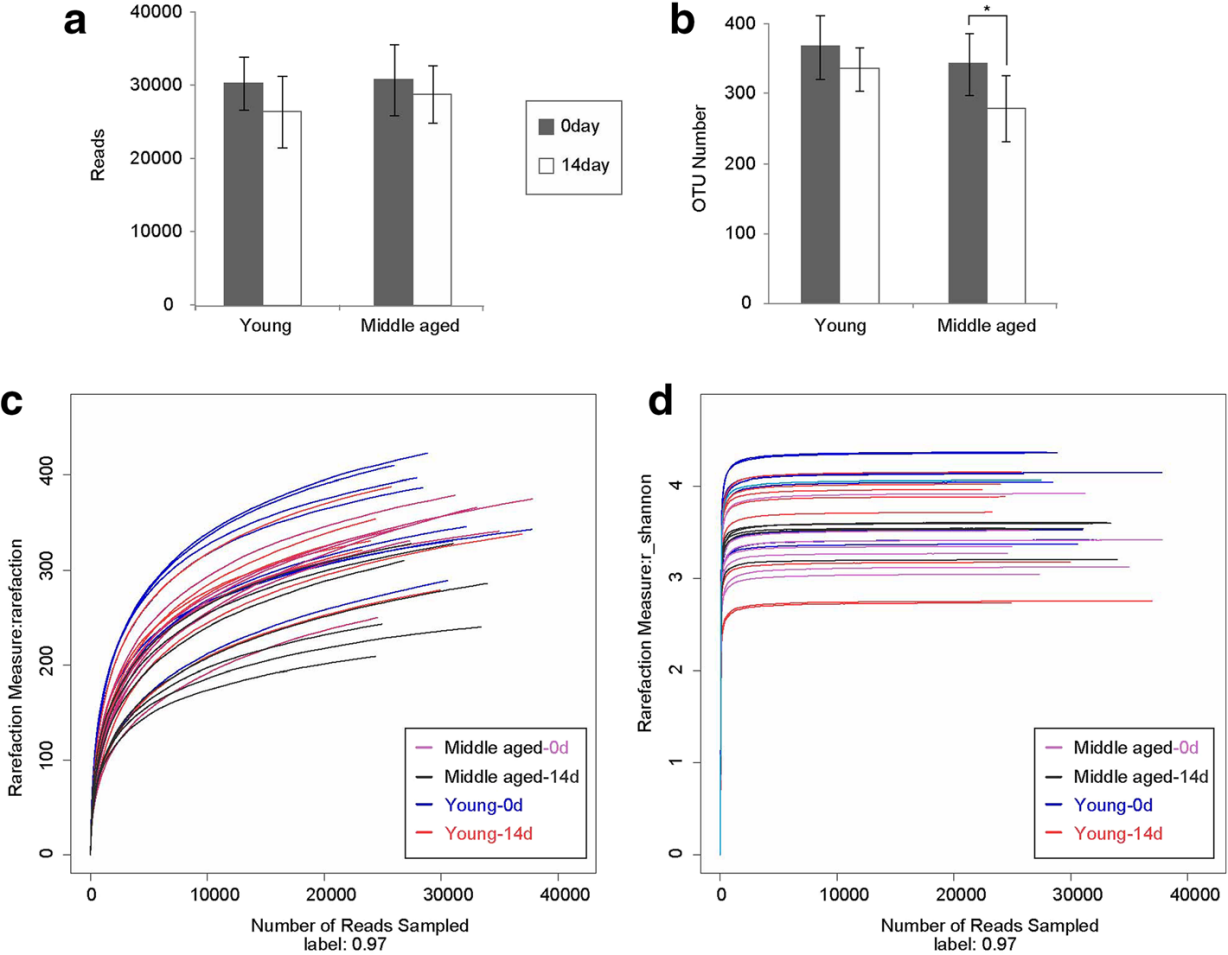
Diversity estimation of gut bacteria in rat feces. **a** Average number of usable raw reads (*bars represent standard deviations*); **b** Average number of OTUs at the 97% similarity level (*bars represent standard deviations*); **c** Rarefaction curves at the 97% similarity level. Each curve represents one biological sample; **d** Shannon–Wiener diversity index curves at the 97% similarity level. Each curve represents one biological sample

There were 871,264 usable raw reads and 9955 species-level operation taxonomy units (OTUs) from all 30 samples. The averages were 29,042 ± 4456 reads and 332 ± 52 OTUs per biological sample (Fig. 1a & b). There was no significant difference in the number of usable raw reads between the diet and age groups (*p* > 0.05). On day 0, the numbers of OTUs did not differ (*p* > 0.05) between the young and middle-aged groups; however, the middle-aged group had fewer OTUs than the young group on day 14 (*p* < 0.05). Although the Rarefaction curves did not reach a stable state (Fig. 1c), the Shannon-Wiener diversity estimates from all the samples became stable (Fig. 1d), indicating that microbial diversity did not change too much during growth. No significant differences (*p* > 0.05) existed between the young and middle-aged groups at the two time points in ACE, Chao, Shannon, Simpson and Good’s coverage indices for gut bacteria (see in Additional file 1: Table S1).

### Overall profiling of gut microbiota

**Fig. 2:**
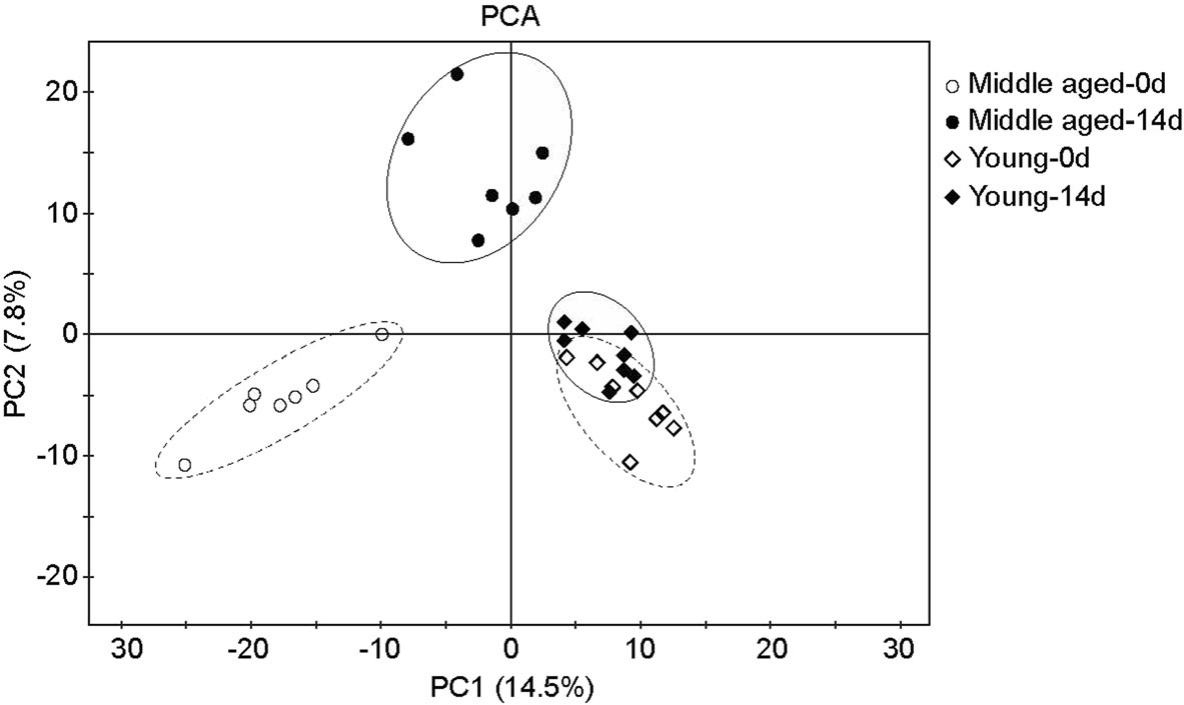
OTU principal component analysis of gut bacteria

Multivariate analyses were performed to discriminate fecal samples of gut bacteria for OTU. Principal component analysis (PCA) showed age-dependent structural rearrangement and a substantial inter- and intra- variation of gut bacteria as a response to diet shift (Fig. 2). The composition of gut bacteria showed differences between young and middle-aged rat samples at two time points. Diet changes resulted in a greater alteration in the composition of gut bacteria for the middle-aged rats; however, it was less pronounced in the young rats.

**Fig. 3:**
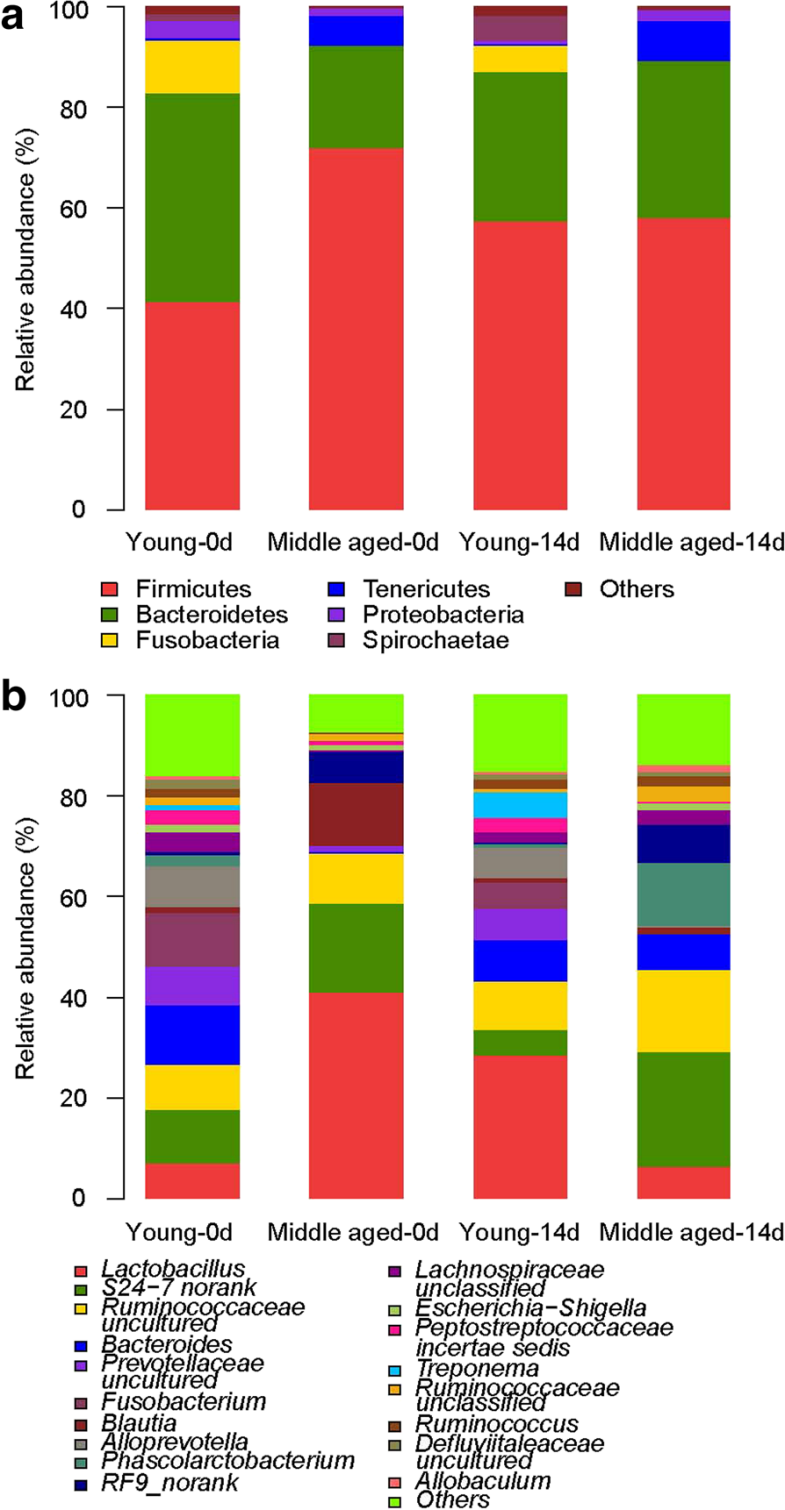
Relative abundances of gut bacteria by phylum and genus. **a** Phylum; **b** Genus

### Composition of gut microbiota

At the phylum level, Firmicutes and Bacteroidetes were the two predominant phyla (Fig. 3a and Table 1). On day 0, Firmicutes accounted for 72.1% ± 11.6% of the total abundance of gut bacteria, while 20.5% ± 12.0% for Bacteroidetes in the middle-aged group. For young rats, the relative abundances of Firmicutes and Bacteroidetes were 41.5% ± 7.9% and 41.3% ± 8.1%, respectively. On day 14, the percentage of Firmicutes decreased to 58.1% ± 18% for the middle-aged rats and increased to 57.7% ± 21.1% for young rats (*p* < 0.01). However, the percentage of Bacteroidetes increased to 31.2% (20.5% on day 0, *p* > 0.05) for the middle-aged rats and decreased to 29.6% for the young rats (41.3% on day 0, *p* > 0.05). This indicated that the baselines of the phyla Firmicutes and Bacteroidetes were quite different and they had opposite responses to the intake of chicken protein. In addition, the young group had higher relative abundances of Fusobacteria compared with the middle-aged group at the two time points (*p* < 0.05, 10.6% versus 0.064% on day 0, and 9.8% versus 0.006% on day 14). Chicken protein intake induced a greater increase in the relative abundance of Spirochaetae in the young group (*p* < 0.05, 0.12% at the baseline versus 4.84% on day 14); however, there was no influence on the middle-aged group (*p* > 0.05, 0.002% on day 0 versus 0.001% on day 14).

**Fig. 4:**
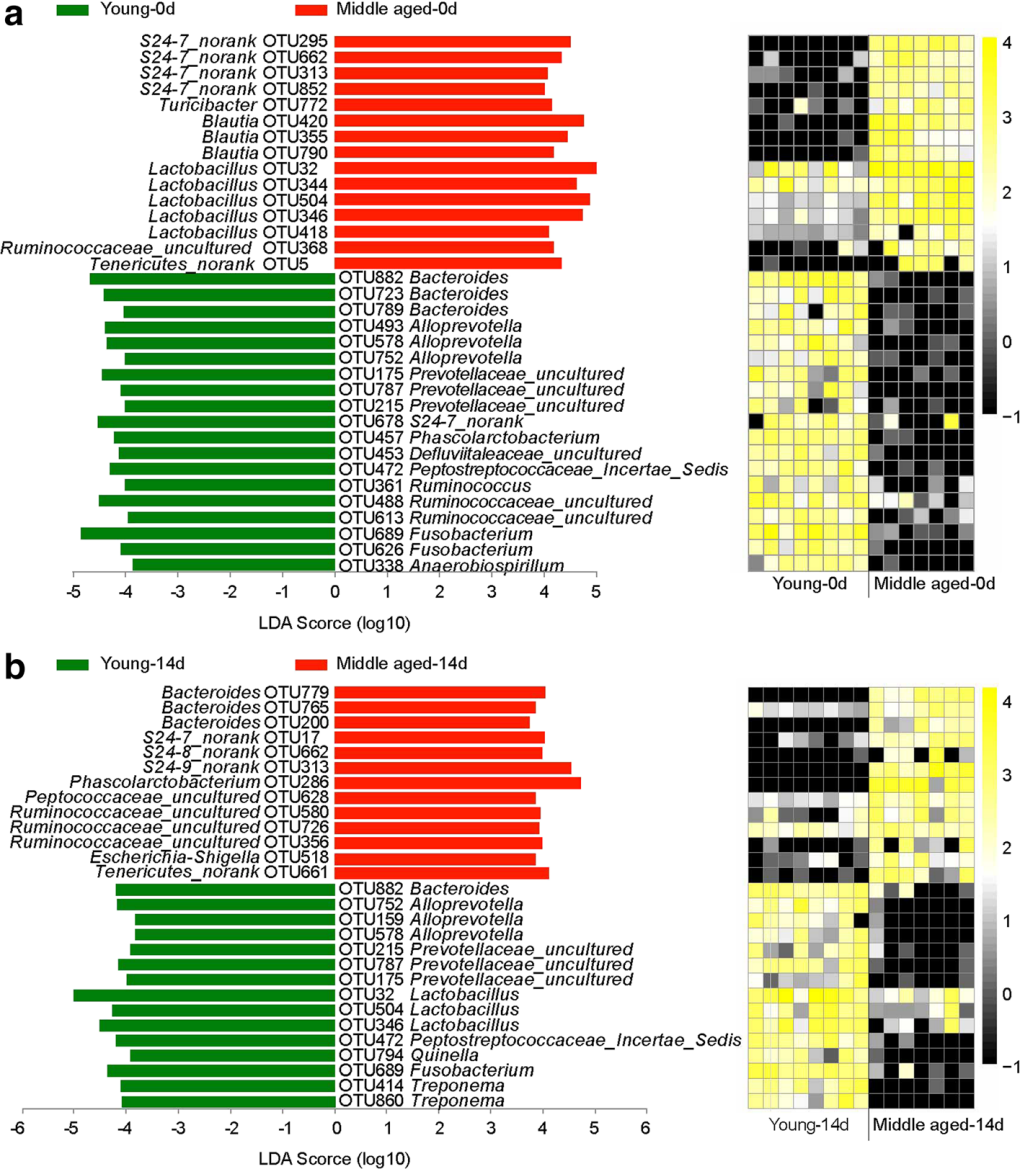
Specific phylotypes of gut bacteria in response to age using LEfSe. **a** Day 0; **b** Day 14. The left histogram shows the LDA scores computed for features at the OTU level. The right heatmap shows the relative abundance of OTU (log 10 transformed). Each column represents one animal and each row represents the OTU corresponding to left one. The color intensity scale showed the relative abundance of OTU (log 10 transformed), *yellow* denotes an high relative abundance of OTU while *black* denotes a low relative abundance of OTU

**Table 1:** Relative abundance of primary phyla in rat feces

	Young-0d	Middle-aged-0d	Young-14d	Middle-aged-14d
Firmicutes	41.48% ± 7.88%	72.07% ± 11.60%	57.66% ± 21.12%	58.11% ± 17.99%
Bacteroidetes	41.28% ± 8.10%	20.47% ± 12.04%	29.61% ± 15.15%	31.18% ± 19.54%
Fusobacteria	10.58% ± 9.84%	0.01% ± 0.01%	5.25% ± 3.72%	0.06% ± 0.16%
Tenericutes	0.61% ± 1.01%	5.85% ± 6.13%	0.25% ± 0.34%	7.99% ± 9.27%
Proteobacteria	3.27% ± 4.12%	1.31% ± 1.45%	0.60% ± 0.40%	2.09% ± 2.00%
Spirochaetae	1.21% ± 0.88%	0.00% ± 0.00%	4.84% ± 5.64%	0.00% ± 0.00%
Other	1.58% ± 1.19%	0.28% ± 0.36%	1.80% ± 2.66%	0.57% ± 0.53%

At the genus level, the composition of gut bacteria was significantly different between the young and middle-aged groups in response to diet and age (Fig. 3b and Table 2). On day 0, the middle-aged group had higher relative abundances of *Lactobacillus*, *S24_7 norank* and *RF9 norank*, and lower relative abundances of *Fusobacterium*, *Alloprevotella*, *Prevotellaceae uncultured*, *Peptostreptococcaceae incertaes*, *Roseburia*, *Treponema*, *Anaerobiospirillum*, *Anaerotruncus* and *Quinella* compared with the young rat group. The relative abundance of *Bacteroides* (11.9% for the young group and 0.43% for the middle-aged group) was lower in the middle-aged group (*p* < 0.001). On day 14, the relative abundance of *Bacteroides* was increased to 7.0% in the middle-aged group (*p* < 0.05) and decreased to 8.0% in the young group. However, the relative abundance of *Lactobacillus* was the opposite, which significantly decreased in the middle-aged group (*p* < 0.001, 40.9% on day 0 versus 6.21% on day 14) and increased in the young group (7.0% on day 0 versus 28.5% on day 14). The middle-aged group had a higher abundance of *Phascolarctobacterium* (*p* < 0.001, 0.001 and 12.2% on days 0 and 14, respectively) after being fed chicken protein. However, no significant change occurred in the young group (*p* > 0.05, 2.1 and 0.67% for days 0 and 14, respectively). Chicken protein diet decreased the relative abundance of *Blautia* in both young and middle-aged rats.

**Table 2:** Relative abundance of primary genera in rat feces

	Young-0d	Middle-aged-0d	Young-14d	Middle-aged-14d
*Lactobacillus*	7.0% ± 8.1%	40.9% ± 14.3%	28.5% ± 24.8%	6.2% ± 10.0%
*S24-7_norank*	10.7% ± 4.3%	17.6% ± 8.7%	5.1% ± 4.1%	22.9% ± 19.3%
*Ruminococcaceae_uncultured*	8.9% ± 5.1%	9.9% ± 6.3%	9.6% ± 10.7%	16.3% ± 6.9%
*Bacteroides*	11.9% ± 6.0%	0.4% ± 0.6%	8.0% ± 4.6%	7.0% ± 4.5%
*Prevotellaceae_uncultured*	7.6% ± 3.1%	1.1% ± 1.1%	6.3% ± 6.5%	0.0% ± 0.1%
*Fusobacterium*	10.6% ± 9.8%	0.0% ± 0.0%	5.2% ± 3.7%	0.1% ± 0.2%
*Blautia*	1.4% ± 2.2%	12.7% ± 12.0%	0.9% ± 0.7%	1.4% ± 1.1%
*Alloprevotella*	8.0% ± 6.5%	0.1% ± 0.1%	6.2% ± 5.7%	0.4% ± 0.8%
*Phascolarctobacterium*	2.1% ± 1.0%	0.0% ± 0.0%	0.7% ± 0.6%	12.2% ± 7.5%
*RF9_norank*	0.6% ± 1.0%	5.8% ± 6.1%	0.2% ± 0.3%	8.0% ± 9.3%
*Lachnospiraceae_unclassified*	4.0% ± 2.5%	0.6% ± 0.4%	1.8% ± 1.2%	2.8% ± 2.5%
*Escherichia-Shigella*	1.4% ± 3.8%	0.8% ± 1.3%	0.0% ± 0.1%	1.3% ± 1.9%
*Peptostreptococcaceae_incertae_sedis*	2.9% ± 3.8%	1.1% ± 0.8%	3.1% ± 4.0%	0.2% ± 0.2%
*Treponema*	1.2% ± 0.9%	0.0% ± 0.0%	4.8% ± 5.6%	0.0% ± 0.0%
*Ruminococcaceae_unclassified*	1.6% ± 1.2%	1.0% ± 0.4%	0.8% ± 0.5%	3.3% ± 3.2%
*Ruminococcus*	1.7% ± 1.8%	0.4% ± 0.3%	2.0% ± 2.0%	1.9% ± 1.0%
*Defluviitaleaceae_uncultured*	1.8% ± 1.7%	0.0% ± 0.0%	0.9% ± 0.4%	1.1% ± 0.7%
*Allobaculum*	0.6% ± 0.2%	0.0% ± 0.0%	0.7% ± 1.2%	1.1% ± 1.4%
Other	0.0% ± 5.4%	0.0% ± 1.5%	0.0% ± 5.2%	0.0% ± 6.5%

### Specific phylotypes varying with age and diet

PCA and ANOVA test revealed changes in the composition of gut microbiota in response to age and diet. To identify specific phylotypes for age and diet, linear discriminant analysis effect size (LEfSe) was performed on the OTU level with a relative abundance that was at least more than 1% in one group.

#### Age effect

At baseline (day 0), 34 OTUs were significantly different between the young and middle-aged groups (Fig. 4a, Additional file 1: Table S2). The relative abundances of 15 OTUs were higher in the middle-aged group, including the genus *Lactobacillus* (OTU32, OTU344, OTU504, OTU346 and OTU418), *S24-7 norank* (OTU295, OTU662, OTU313 and OTU852) and *Blautia* (OTU420, OTU355 and OTU790). However, 19 OTUs were more abundant in the young group, including the genus *Bacteroides* (OTU882, OTU723 and OTU789) and *Fusobacterium* (OTU 689 and OTU626).

**Fig. 5:**
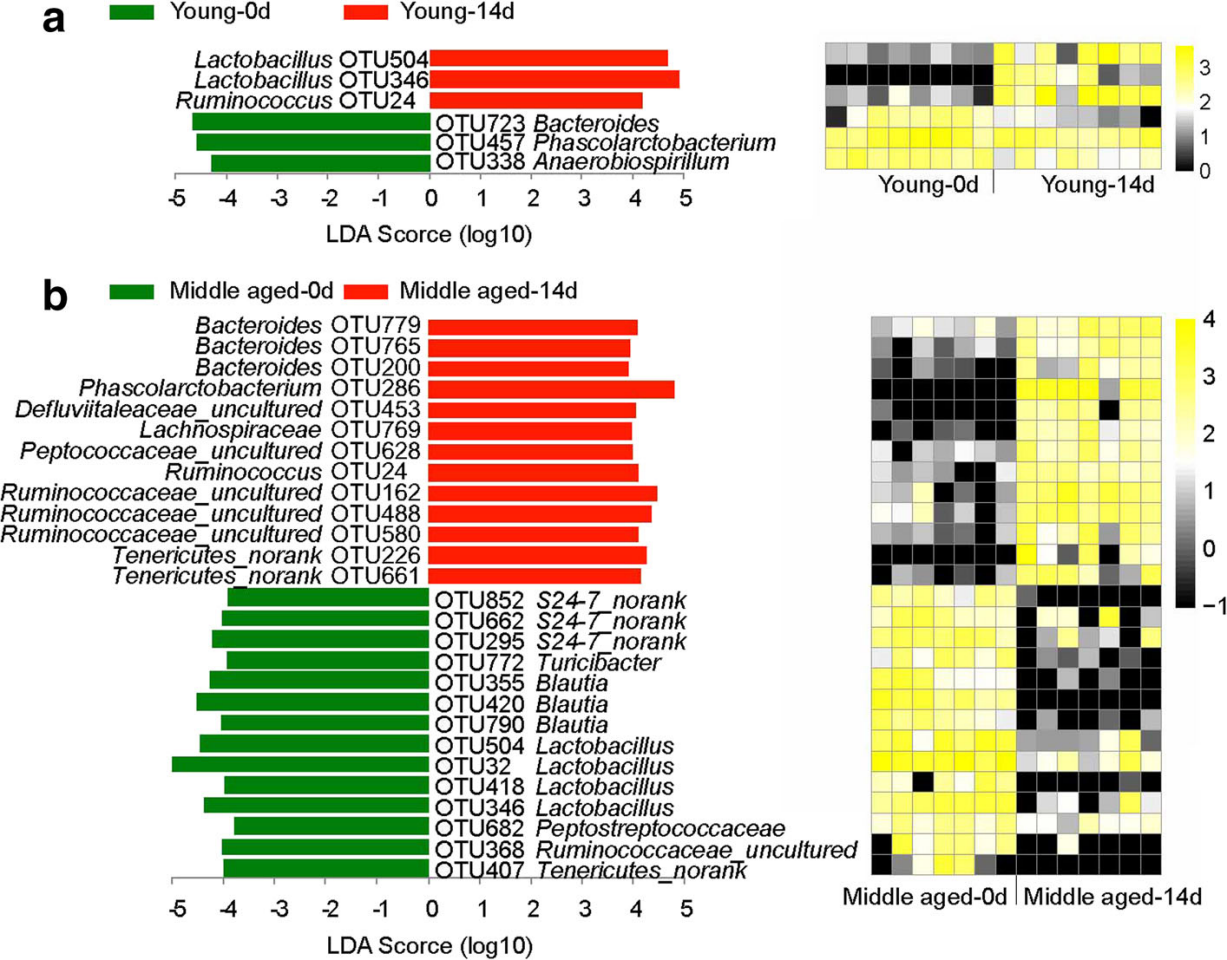
Specific phylotypes of gut bacteria in response to diet shift using LEfSe. **a** Young group; **b** Middle-aged group. The left histogram shows the LDA scores computed for features at the OTU level. The right heatmap shows the relative abundance of OTU (log 10 transformed). Each column represents one animal and each row represents the OTU corresponding to left one. The color intensity scale showed the relative abundance of OTU (log 10 transformed), *yellow* denotes an high relative abundance of OTU while *black* denotes a low relative abundance of OTU

On day 14, 28 OTUs were significantly different between the middle-aged and young groups (*p* < 0.05, Fig. 4b, Additional file 1: Table S3). Thirteen OTUs had higher relative abundances in the middle-aged group, including the genera *S24-7 norank*, *Lactobacillus* and *Blautia*. The other 15 OTUs were highly abundant in the young group, including the genera *Fusobacterium*, *Alloprevotella*, and *Prevotellaceae_uncultured*.

#### Diet effect

For the young rats, only 6 OTUs showed significant responses to diet (Fig. 5a, Additional file 1: Table S4). The relative abundances of three OTUs increased with the diet shift, including the genera *Lactobacillus* (OTU504 and OTU346) and *Ruminococcus* (OTU24). The other three OTUs showed negative responses to the diet shift, including the genera *Bacteroides* (OTU723), *Phascolarctobacterium* (OTU457), and *Anaerobiospirillum* (OTU338).

For the middle-aged rats, 27 OTUs had positive or negative responses to the diet change (Fig. 5b, Additional file 1: Table S5). Thirteen of them were positive, including the genus *Bacteroides* (OTU779, OTU765, OTU200) and several other genera. Eighteen were negative, including the genera *Lactobacillus* (OTU504, OTU32, OTU418 and OTU346), *S24-7 norank* (OTU852, OTU662, OTU295) and *Blautia* (OTU355, OTU420, OTU790).

## Discussion

The ratio of the phylum *Firmicutes* to the phylum *Bacteroidetes* could be associated with obesity [14]. This ratio showed a positive response to diet change from normal chow (17.7% casein diet) to formulated chicken protein diet for the young group, but a negative response was observed for the middle-aged rats. However, none of rats showed obese characteristics. This could be attributed to two aspects: (1) chicken protein in the diet may down regulate lipid metabolism pathway in liver [15]; (2) it is specific bacteria strains but not the phyla that contribute to obesity and other physiological responses [16]. For example, the *Lactobacillus gasseri* strain has an anti-obesity effect in overweight and obese people [14]. Moreover, some studies even showed lower ratios of the phylum *Firmicutes* to the phylum *Bacteroidetes* in obese individuals [17, 18]. *Lactobacilli* have the ability to convert lactose and other sugars into lactic acid and have been used to treat and prevent diarrhea [19]. In addition, *Lactobacillus* plays an important role in host health and immune function due to their potential to inhibit the growth of pathogens and to prevent intestinal disorders [20]. Therefore, the intake of chicken protein might decrease the functions of *Lactobacillus* in middle-aged rats. However, other aspects should be considered. For example, serum lipopolysaccharide binding protein is usually considered a biomarker for an inflammatory response and an antigen load to the host [21]. This needs to be further studied.

*Bacteroides* have genes encoding hydrolases of soluble polysaccharides and have the capability of utilizing various substrates [22, 23]. *Firmicutes* typically carry fewer genes for polysaccharide degradation [24]. However, a previous fermentation study showed that human gut bacteria could degrade insoluble starch particles and wheat bran, and these bacteria belonged to the phylum *Firmicutes* but not the phylum *Bacteroides* [23]. The species *Ruminococcus bromii*, in the phylum *Firmicutes*, plays a key role in the degradation of resistant starch [25]. In fact, the genes encoding hydrolases for polysaccharide degradation in *Firmicutes* were less studied than those in *Bacteroides*. Therefore, it is difficult to associate the relative abundance of phylotypes in the genus *Bacteroides* with the level of polysaccharide degradation. It needs further study.

Changes in the composition of gut microbiota could be one of age-related physiological processes. Previous studies showed that a high similarity of the gut bacterial community in the elderly to that of younger adults indicated a high probability for the elderly to be relatively healthy [26]. However, it could be more important for the host to have the ability to establish a new balance for the age-related bacteria. In the study, gut microbiota showed different responses to the intake of chicken protein between young and middle-aged rats. A formulated chicken protein diet (protein content: 17.8%) caused a reduction in the phylum *Firmicutes* and an increase in the *Bacteroides* for middle-aged rats, with the opposite results for young rats. In addition, the relative abundance of the genus *Lactobacillus* increased for young rats after they were fed chicken protein for 14 days, which may reduce the antigen load and inflammatory response from gut bacteria to the host. However, the abundance of the genus *Lactobacillus* decreased in middle-aged rats, which may affect host health.

Finally, the capacities of protein digestion and absorption in the small intestine may different between young and middle ages [27]. This would cause more undigested or unabsorbed dietary proteins to enter into the large intestine and alter the gut bacteria composition.

## Conclusion

Our results showed that gut microbiota could be changed by 17.7% chicken protein diet and the effects were significantly different between middle-aged and young rats. Chicken protein intake promoted the beneficial genus *Lactobacillus* in young rats, but an opposite effect was observed in the middle-aged group. The association between meat protein intake and gut microbiota in middle-aged rats needs to be further studied. Meanwhile, to evaluate the association between diet and host health, age effect should be considered in future studies.

## Additional file

Additional file 1:
**Table S1.** Richness and diversity indexes relative to each sample. **Table S2.** The differentially fecal bacterial communities between Young-0d and Middle aged-0d using LEfSe at the OTU level. **Table S3.** The differentially fecal bacterial communities between Young-14d and Middle aged-14d using LEfSe at the OTU level. **Table S4.** The differentially fecal bacterial communities between Young-0d and Young-14d using LEfSe at the OTU level. **Table S5.** The differentially fecal bacterial communities between Middle aged-0d and Middle aged-14d using LEfSe at the OTU level. (DOC 254 kb)

## Abbreviations

ANOVA: Analysis of variance
LefSe: Linear discriminant analysis effect size
OTU: Operation taxonomy units
PCA: Principal component analysis

## References

[1] O’Hara AM, Shanahan F. The gut flora as a forgotten organ. EMBO Rep. 2006;7(7):688–693. 10.1038/sj.embor.7400731 PMC1500832 16819463

[2] Kleessen B, Sykura B, Zunft HJ, Blaut M. Effects of inulin and lactose on fecal microflora, microbial activity, and bowel habit in elderly constipated persons. Am J Clin Nutr. 1997;65(5):1397–1402. 10.1093/ajcn/65.5.1397 9129468

[3] Franceschi C, Bonafè M, Valensin S, Olivieri F, de Luca M, Ottaviani E, et al. Inflamm‐aging: an evolutionary perspective on immunosenescence. Ann N Y Acad Sci. 2000;908(1):244–254. 10.1111/j.1749-6632.2000.tb06651.x 10911963

[4] Salminen A, Huuskonen J, Ojala J, Kauppinen A, Kaarniranta K, Suuronen T (2008). Activation of innate immunity system during aging: NF-kB signaling is the molecular culprit of inflamm-aging. Ageing Res Rev.

[5] Guigoz Y, Doré J, Schiffrin EJ (2008). The inflammatory status of old age can be nurtured from the intestinal environment. Curr Opin Clin Nutr.

[6] Reber AJ, Chirkova T, Kim JH, Cao W, Biber R, Shay DK (2012). Immunosenescence and challenges of vaccination against influenza in the aging population. Aging Dis.

[7] Gilbert JA, Bendsen NT, Tremblay A, Astrup A (2011). Effect of proteins from different sources on body composition. Nutr Metab Cardiovasc Dis.

[8] Schulze MB, Manson JE, Willett WC, Hu FB. Processed meat intake and incidence of Type 2 diabetes in younger and middle-aged women. Diabetologia. 2003;46(11):1465–1473. 10.1007/s00125-003-1220-7 14576980

[9] Chen J, Stampfer MJ, Hough HL, Garcia-Closas M, Willett WC, Hennekens CH, Kelsey KT, Hunter DJ. A prospective study of N-acetyltransferase genotype, red meat intake, and risk of colorectal cancer. Cancer Res. 1998;58(15):3307–3311. 9699660

[10] Zhu Y, Lin X, Zhao F, Shi X, Li H, Li Y, et al. Meat, dairy and plant proteins alter bacterial composition of rat gut bacteria. Sci Rep. 2015;5:15220. doi:10.1038/srep15220. 10.1038/srep15220 PMC4604471 26463271

[11] Flurkey KM, Currer J, Harrison DE. Chapter 20 - mouse models in aging research A2 - Fox, James G. In: Davisson MT, Quimby FW, Barthold SW, Newcomer CE, Smith AL, editors. The mouse in biomedical research. 2nd ed. Burlington: Academic; 2007. p. 637–672.

[12] Reeves PG, Nielsen FH, Fahey GC. AIN-93 purified diets for laboratory rodents: final report of the American Institute of Nutrition ad hoc writing committee on the reformulation of the AIN-76A rodent diet. J Nutr. 1993;123(11):1939–1951. 10.1093/jn/123.11.1939 8229312

[13] Segata N, Izard J, Waldron L, Gevers D, Miropolsky L, Garrett WS, et al. Metagenomic biomarker discovery and explanation. Genome Biol. 2011;12:R60. doi: 10.1186/gb-2011-12-6-r60. 10.1186/gb-2011-12-6-r60 PMC3218848 21702898

[14] Million M, Lagier JC, Yahav D, Paul M. Gut bacterial microbiota and obesity. Clin Microbiol Infect. 2013;19(4):305–313. 10.1111/1469-0691.12172 23452229

[15] Song S, Hooiveld GJEJ, Li M, Zhao F, Zhang W, Xu X, et al. Distinct physiological, plasma amino acid, and liver transcriptome responses to purified dietary beef, chicken, fish, and pork proteins in young rats. Mol Nutr Food Res. 2016;60:1199–1205. 10.1002/mnfr.201500789 26833809

[16] Zhao L. The gut microbiota and obesity: from correlation to causality. Nat Rev Microbiol. 2013;11:639–647. 10.1038/nrmicro3089 23912213

[17] Schwiertz A, Taras D, Schafer K, Beijer S, Bos NA, Donus C, et al. Microbiota and SCFA in lean and overweight healthy subjects. Obesity. 2010;18(1):190–195. 10.1038/oby.2009.167 19498350

[18] Collado MC, Isolauri E, Laitinen K, Salminen S. Distinct composition of gut microbiota during pregnancy in overweight and normal-weight women. Am J Clin Nutr. 2008;88(4):894–899. 10.1093/ajcn/88.4.894 18842773

[19] Lee BJ, Bak YT. Irritable bowel syndrome, gut microbiota and probiotics. J Neurogastroenterol Motil. 2011;17(3):252–266. 10.5056/jnm.2011.17.3.252 PMC3155061 21860817

[20] Marco ML, de Vries MC, Wels M, Molenaar D, Mangell P, Ahrne S, et al. Convergence in probiotic Lactobacillus gut-adaptive responses in humans and mice. ISME J. 2010;4(11):1481–1484. 10.1038/ismej.2010.61 20505752

[21] Zweigner J, Schumann RR, Weber JR. The role of lipopolysaccharide-binding protein in modulating the innate immune response. Microbes Infect. 2006;8(3):946–952. 10.1016/j.micinf.2005.10.006 16483818

[22] Martens EC, Koropatkin NM, Smith TJ, Gordon JI. Complex glycan catabolism by the human gut microbiota: the Bacteroidetes Sus-like paradigm. J Biol Chem. 2009;284(37):24673–24677. 10.1074/jbc.R109.022848 PMC2757170 19553672

[23] Leitch EC, Walker AW, Duncan SH, Holtrop G, Flint HJ. Selective colonization of insoluble substrates by human faecal bacteria. Environ Microbiol. 2007;9(3):667–679. 10.1111/j.1462-2920.2006.01186.x 17298367

[24] Kaoutari AE, Armougom F, Gordon JI, Raoult D, Henrissat B (2013). The abundance and variety of carbohydrate-active enzymes in the human gut microbiota. Nat Rev Microbiol.

[25] Ze X, Duncan SH, Louis P, Flint HJ. Ruminococcus bromii is a keystone species for the degradation of resistant starch in the human colon. ISME J. 2012;6(8):1535–1543. 10.1038/ismej.2012.4 PMC3400402 22343308

[26] Biagi E, Candela M, Turroni S, Garagnani P, Franceschi C, Brigidi P (2013). Ageing and gut microbes: perspectives for health maintenance and longevity. Pharmacol Res.

[27] Paddon-Jones D, Campbell WW, Jacques PF, Kritchevsky SB, Moore LL, Rodriguez NR, et al. Protein and healthy aging. Am J Clin Nutr. 2015;101(6):1339S–1345S. 10.3945/ajcn.114.084061 25926511

