# Supplementary material for: The gut microbiota in young and middle-aged rats showed different responses to chicken protein in their diet
Source: BMC Microbiol. 2016 Nov 25;16:281. doi: 10.1186/s12866-016-0895-0 (PMC5124274; doi:10.1186/s12866-016-0895-0)
Supplement: Additional file 1: — Table S1. Richness and diversity indexes relative to each sample. Table S2. The differentially fecal bacterial communities between Young-0d and Middle aged-0d using LEfSe at the OTU level. Table S3. The differentially fecal bacterial communities between Young-14d and Middle aged-14d using LEfSe at the OTU level. Table S4. The differentially fecal bacterial communities between Young-0d and Young-14d using LEfSe at the OTU level. Table S5. The differentially fecal bacterial communities between Middle aged-0d and Middle aged-14d using LEfSe at the OTU level. (DOC 254 kb) [file 12866_2016_895_MOESM1_ESM.doc]

**Supplementary Table S1 Richness and diversity indexes relative to each sample.**

| Sample ID | Reads | 0.97 | | | | | |
| --- | --- | --- | --- | --- | --- | --- | --- |
|  |  | OTU  Number | ACE | Chao | Coverage | Shannon | Simpson |
| Young-0d-1 | 28827 | 424 | 481 | 492 | 0.997 | 4.36 | 0.0314 |
|  |  |  | (460,514) | (462,545) |  | (4.34,4.38) | (0.0305,0.0322) |
| Young-0d-2 | 28416 | 388 | 435 | 460 | 0.998 | 4.05 | 0.0493 |
|  |  |  | (416,465) | (426,522) |  | (4.03,4.07) | (0.0479,0.0506) |
| Young-0d-3 | 37758 | 344 | 394 | 428 | 0.998 | 4.15 | 0.0286 |
|  |  |  | (374,429) | (387,509) |  | (4.13,4.16) | (0.0281,0.029) |
| Young-0d-4 | 30978 | 332 | 378 | 395 | 0.998 | 3.53 | 0.0779 |
|  |  |  | (359,409) | (365,455) |  | (3.51,3.55) | (0.0763,0.0795) |
| Young-0d-5 | 30555 | 290 | 367 | 398 | 0.997 | 3.37 | 0.0651 |
|  |  |  | (338,413) | (350,487) |  | (3.36,3.39) | (0.0641,0.0662) |
| Young-0d-6 | 27916 | 398 | 449 | 456 | 0.998 | 4.37 | 0.0297 |
|  |  |  | (429,481) | (429,506) |  | (4.35,4.39) | (0.0289,0.0305) |
| Young-0d-7 | 25967 | 411 | 468 | 465 | 0.997 | 4.13 | 0.0408 |
|  |  |  | (447,501) | (441,508) |  | (4.11,4.15) | (0.0397,0.0418) |
| Young-0d-8 | 32154 | 347 | 408 | 420 | 0.998 | 3.42 | 0.096 |
|  |  |  | (385,446) | (387,482) |  | (3.4,3.44) | (0.094,0.0981) |
| Middle aged-0d-1 | 34960 | 342 | 382 | 392 | 0.998 | 3.12 | 0.144 |
|  |  |  | (365,410) | (367,439) |  | (3.1,3.15) | (0.1411,0.1469) |
| Middle aged-0d-2 | 26379 | 345 | 412 | 424 | 0.997 | 3.52 | 0.0629 |
|  |  |  | (388,451) | (389,486) |  | (3.5,3.54) | (0.0617,0.0641) |
| Middle aged-0d-3 | 31185 | 380 | 447 | 461 | 0.997 | 3.92 | 0.0474 |
|  |  |  | (423,487) | (426,525) |  | (3.9,3.94) | (0.0464,0.0484) |
| Middle aged-0d-4 | 24549 | 251 | 327 | 305 | 0.997 | 3.27 | 0.0732 |
|  |  |  | (298,374) | (280,351) |  | (3.25,3.29) | (0.0718,0.0746) |
| Middle aged-0d-5 | 33046 | 367 | 488 | 504 | 0.997 | 3.61 | 0.0574 |
|  |  |  | (448,548) | (449,597) |  | (3.59,3.63) | (0.0564,0.0584) |
| Middle aged-0d-6 | 37810 | 376 | 448 | 459 | 0.998 | 3.42 | 0.1143 |
|  |  |  | (422,489) | (423,523) |  | (3.4,3.44) | (0.1116,0.117) |
| Middle aged-0d-7 | 27317 | 332 | 411 | 399 | 0.997 | 3.05 | 0.1509 |
|  |  |  | (383,456) | (370,450) |  | (3.02,3.07) | (0.1473,0.1545) |
| Young-14d-1 | 25744 | 389 | 449 | 458 | 0.997 | 4.15 | 0.032 |
|  |  |  | (427,485) | (427,514) |  | (4.14,4.17) | (0.0314,0.0327) |
| Young-14d-2 | 24894 | 342 | 397 | 398 | 0.997 | 2.74 | 0.2455 |
|  |  |  | (376,431) | (373,444) |  | (2.71,2.77) | (0.2401,0.2509) |
| Young-14d-3 | 22392 | 318 | 370 | 372 | 0.997 | 3.97 | 0.0341 |
|  |  |  | (349,404) | (347,420) |  | (3.95,3.99) | (0.0334,0.0347) |
| Young-14d-4 | 23238 | 322 | 376 | 369 | 0.997 | 3.72 | 0.0643 |
|  |  |  | (355,410) | (347,409) |  | (3.7,3.74) | (0.0626,0.0659) |
| Young-14d-5 | 36936 | 339 | 395 | 395 | 0.998 | 2.75 | 0.2133 |
|  |  |  | (374,429) | (370,441) |  | (2.73,2.78) | (0.2093,0.2174) |
| Young-14d-6 | 29914 | 280 | 344 | 351 | 0.998 | 3.18 | 0.0969 |
|  |  |  | (319,384) | (318,412) |  | (3.16,3.2) | (0.0949,0.0989) |
| Young-14d-7 | 24369 | 355 | 428 | 423 | 0.997 | 3.89 | 0.0423 |
|  |  |  | (402,469) | (394,476) |  | (3.87,3.91) | (0.0413,0.0432) |
| Young-14d-8 | 23956 | 332 | 371 | 382 | 0.998 | 4.03 | 0.0385 |
|  |  |  | (355,399) | (358,429) |  | (4.01,4.05) | (0.0376,0.0395) |
| Middle aged-14d-1 | 24948 | 244 | 290 | 291 | 0.998 | 3.35 | 0.0972 |
|  |  |  | (271,325) | (267,339) |  | (3.33,3.37) | (0.0946,0.0998) |
| Middle aged-14d-2 | 33968 | 287 | 349 | 363 | 0.998 | 3.21 | 0.0883 |
|  |  |  | (325,390) | (328,428) |  | (3.19,3.22) | (0.0868,0.0898) |
| Middle aged-14d-3 | 31034 | 329 | 388 | 415 | 0.998 | 3.54 | 0.0756 |
|  |  |  | (365,425) | (376,489) |  | (3.52,3.56) | (0.074,0.0772) |
| Middle aged-14d-4 | 33421 | 241 | 274 | 264 | 0.999 | 3.6 | 0.0495 |
|  |  |  | (259,302) | (251,291) |  | (3.58,3.62) | (0.0487,0.0503) |
| Middle aged-14d-5 | 24392 | 210 | 248 | 249 | 0.998 | 3.55 | 0.0669 |
|  |  |  | (231,281) | (228,295) |  | (3.53,3.57) | (0.0649,0.0688) |
| Middle aged-14d-6 | 26810 | 311 | 390 | 405 | 0.997 | 3.54 | 0.0815 |
|  |  |  | (361,437) | (363,481) |  | (3.51,3.56) | (0.0793,0.0837) |
| Middle aged-14d-7 | 27431 | 329 | 387 | 423 | 0.997 | 4.07 | 0.0389 |
|  |  |  | (364,424) | (379,505) |  | (4.05,4.08) | (0.0379,0.0399) |

**Supplementary Table S2 The differentially fecal bacterial communities between** **Young-0d and Middle aged-0d using LEfSe at the OTU level**.

| OTU ID | Toxon | | | Riched class | LDA Score (log10) | p value |
| --- | --- | --- | --- | --- | --- | --- |
|  | Phylum | Family | Genus |  |  |  |
| OTU453 | Firmicutes | Defluviitaleaceae | uncultured | Young-0d | 3.87 | <0.001 |
| OTU882 | Bacteroidetes | Bacteroidaceae | Bacteroides | Young-0d | 4.37 | <0.001 |
| OTU578 | Bacteroidetes | Prevotellaceae | Alloprevotella | Young-0d | 4.08 | <0.001 |
| OTU457 | Firmicutes | Acidaminococcaceae | Phascolarctobacterium | Young-0d | 3.93 | <0.001 |
| OTU472 | Firmicutes | Peptostreptococcaceae | Incertae_Sedis | Young-0d | 4.01 | <0.001 |
| OTU626 | Fusobacteria | Fusobacteriaceae | Fusobacterium | Young-0d | 3.84 | <0.05 |
| OTU175 | Bacteroidetes | Prevotellaceae | uncultured | Young-0d | 4.16 | <0.05 |
| OTU493 | Bacteroidetes | Prevotellaceae | Alloprevotella | Young-0d | 4.09 | <0.05 |
| OTU787 | Bacteroidetes | Prevotellaceae | uncultured | Young-0d | 3.82 | <0.05 |
| OTU338 | Proteobacteria | Succinivibrionaceae | Anaerobiospirillum | Young-0d | 3.62 | <0.05 |
| OTU488 | Firmicutes | Ruminococcaceae | uncultured | Young-0d | 4.22 | <0.05 |
| OTU723 | Bacteroidetes | Bacteroidaceae | Bacteroides | Young-0d | 4.12 | <0.05 |
| OTU752 | Bacteroidetes | Prevotellaceae | Alloprevotella | Young-0d | 3.74 | <0.05 |
| OTU215 | Bacteroidetes | Prevotellaceae | uncultured | Young-0d | 3.74 | <0.05 |
| OTU689 | Fusobacteria | Fusobacteriaceae | Fusobacterium | Young-0d | 4.54 | <0.05 |
| OTU613 | Firmicutes | Ruminococcaceae | uncultured | Young-0d | 3.68 | <0.05 |
| OTU361 | Firmicutes | Ruminococcaceae | Ruminococcus | Young-0d | 3.74 | <0.05 |
| OTU789 | Bacteroidetes | Bacteroidaceae | Bacteroides | Young-0d | 3.77 | <0.05 |
| OTU678 | Bacteroidetes | S24-7 | norank | Young-0d | 4.24 | <0.05 |
| OTU295 | Bacteroidetes | S24-7 | norank | Middle aged-0d | 4.21 | <0.001 |
| OTU852 | Bacteroidetes | S24-7 | norank | Middle aged-0d | 3.74 | <0.001 |
| OTU420 | Firmicutes | Lachnospiraceae | Blautia | Middle aged-0d | 4.44 | <0.001 |
| OTU790 | Firmicutes | Lachnospiraceae | Blautia | Middle aged-0d | 3.92 | <0.001 |
| OTU355 | Firmicutes | Lachnospiraceae | Blautia | Middle aged-0d | 4.16 | <0.001 |
| OTU662 | Bacteroidetes | S24-7 | norank | Middle aged-0d | 4.05 | <0.001 |
| OTU313 | Bacteroidetes | S24-7 | norank | Middle aged-0d | 3.80 | <0.001 |
| OTU504 | Firmicutes | Lactobacillaceae | Lactobacillus | Middle aged-0d | 4.55 | <0.05 |
| OTU346 | Firmicutes | Lactobacillaceae | Lactobacillus | Middle aged-0d | 4.42 | <0.05 |
| OTU772 | Firmicutes | Erysipelotrichaceae | Turicibacter | Middle aged-0d | 3.87 | <0.05 |
| OTU368 | Firmicutes | Ruminococcaceae | uncultured | Middle aged-0d | 3.91 | <0.05 |
| OTU418 | Firmicutes | Lactobacillaceae | Lactobacillus | Middle aged-0d | 3.84 | <0.05 |
| OTU5 | Tenericutes | norank | norank | Middle aged-0d | 4.04 | <0.05 |
| OTU344 | Firmicutes | Lactobacillaceae | Lactobacillus | Middle aged-0d | 4.32 | <0.05 |
| OTU32 | Firmicutes | Lactobacillaceae | Lactobacillus | Middle aged-0d | 4.67 | <0.05 |

**Supplementary Table S3** **The differentially fecal bacterial communities between Young-14d and Middle aged-14d using LEfSe at the OTU level.**

| OTU ID | Toxon | | | Riched class | LDA Score (log10) | p value |
| --- | --- | --- | --- | --- | --- | --- |
|  | Phylum | Family | Genus |  |  |  |
| OTU779 | Bacteroidetes | Bacteroidaceae | Bacteroides | Middle aged-14d | 4.08 | <0.001 |
| OTU200 | Bacteroidetes | Bacteroidaceae | Bacteroides | Middle aged-14d | 3.78 | <0.001 |
| OTU313 | Bacteroidetes | S24-7 | norank | Middle aged-14d | 4.59 | <0.001 |
| OTU286 | Firmicutes | Acidaminococcaceae | Phascolarctobacterium | Middle aged-14d | 4.75 | <0.001 |
| OTU17 | Bacteroidetes | S24-7 | norank | Middle aged-14d | 4.04 | <0.05 |
| OTU628 | Firmicutes | Peptococcaceae | uncultured | Middle aged-14d | 3.90 | <0.05 |
| OTU765 | Bacteroidetes | Bacteroidaceae | Bacteroides | Middle aged-14d | 3.88 | <0.05 |
| OTU661 | Tenericutes | norank | norank | Middle aged-14d | 4.13 | <0.05 |
| OTU518 | Proteobacteria | Enterobacteriaceae | Escherichia-Shigella | Middle aged-14d | 3.89 | <0.05 |
| OTU580 | Firmicutes | Ruminococcaceae | uncultured | Middle aged-14d | 4.00 | <0.05 |
| OTU356 | Firmicutes | Ruminococcaceae | uncultured | Middle aged-14d | 4.02 | <0.05 |
| OTU726 | Firmicutes | Ruminococcaceae | uncultured | Middle aged-14d | 3.95 | <0.05 |
| OTU662 | Bacteroidetes | S24-7 | norank | Middle aged-14d | 4.01 | <0.05 |
| OTU860 | Spirochaetae | Spirochaetaceae | Treponema | Young-14d | 4.08 | <0.001 |
| OTU752 | Bacteroidetes | Prevotellaceae | Alloprevotella | Young-14d | 4.10 | <0.001 |
| OTU414 | Spirochaetae | Spirochaetaceae | Treponema | Young-14d | 4.07 | <0.001 |
| OTU787 | Bacteroidetes | Prevotellaceae | uncultured | Young-14d | 4.10 | <0.001 |
| OTU175 | Bacteroidetes | Prevotellaceae | uncultured | Young-14d | 3.94 | <0.001 |
| OTU472 | Firmicutes | Peptostreptococcaceae | Incertae_Sedis | Young-14d | 4.15 | <0.001 |
| OTU882 | Bacteroidetes | Bacteroidaceae | Bacteroides | Young-14d | 4.16 | <0.05 |
| OTU689 | Fusobacteria | Fusobacteriaceae | Fusobacterium | Young-14d | 4.33 | <0.05 |
| OTU578 | Bacteroidetes | Prevotellaceae | Alloprevotella | Young-14d | 3.79 | <0.05 |
| OTU794 | Firmicutes | Veillonellaceae | Quinella | Young-14d | 3.88 | <0.05 |
| OTU159 | Bacteroidetes | Prevotellaceae | Alloprevotella | Young-14d | 3.76 | <0.05 |
| OTU215 | Bacteroidetes | Prevotellaceae | uncultured | Young-14d | 3.89 | <0.05 |
| OTU32 | Firmicutes | Lactobacillaceae | Lactobacillus | Young-14d | 4.99 | <0.05 |
| OTU346 | Firmicutes | Lactobacillaceae | Lactobacillus | Young-14d | 4.50 | <0.05 |
| OTU504 | Firmicutes | Lactobacillaceae | Lactobacillus | Young-14d | 4.21 | <0.05 |

**Supplementary Table S4 The differentially fecal bacterial communities** **between Young-0d and Young-14d using LEfSe at the OTU level.**

| OTU ID | Toxon | | | Riched class | LDA Score (log10) | p value |
| --- | --- | --- | --- | --- | --- | --- |
|  | Phylum | Family | Genus |  |  |  |
| OTU457 | Firmicutes | Acidaminococcaceae | Phascolarctobacterium | Young-0d | 4.23 | <0.05 |
| OTU723 | Bacteroidetes | Bacteroidaceae | Bacteroides | Young-0d | 4.31 | <0.05 |
| OTU338 | Proteobacteria | Succinivibrionaceae | Anaerobiospirillum | Young-0d | 4.09 | <0.05 |
| OTU518 | Proteobacteria | Enterobacteriaceae | Escherichia-Shigella | Young-0d | 4.12 | <0.05 |
| OTU882 | Bacteroidetes | Bacteroidaceae | Bacteroides | Young-0d | 4.36 | <0.05 |
| OTU24 | Firmicutes | Ruminococcaceae | Ruminococcus | Young-14d | 4.04 | <0.05 |
| OTU346 | Firmicutes | Lactobacillaceae | Lactobacillus | Young-14d | 4.59 | <0.05 |
| OTU504 | Firmicutes | Lactobacillaceae | Lactobacillus | Young-14d | 4.35 | <0.05 |

**Supplementary Table S5 The differentially fecal bacterial communities between Middle aged-0d and Middle aged-14d using LEfSe at the OTU level.**

| OTU ID | Toxon | | | Riched class | LDA Score (log10) | p value |
| --- | --- | --- | --- | --- | --- | --- |
|  | Phylum | Family | Genus |
| OTU420 | Firmicutes | Lachnospiraceae | Blautia | Middle aged-0d | 4.51 | <0.001 |
| OTU852 | Bacteroidetes | S24-7 | norank | Middle aged-0d | 3.90 | <0.05 |
| OTU355 | Firmicutes | Lachnospiraceae | Blautia | Middle aged-0d | 4.25 | <0.05 |
| OTU790 | Firmicutes | Lachnospiraceae | Blautia | Middle aged-0d | 4.02 | <0.05 |
| OTU772 | Firmicutes | Erysipelotrichaceae | Turicibacter | Middle aged-0d | 3.92 | <0.05 |
| OTU32 | Firmicutes | Lactobacillaceae | Lactobacillus | Middle aged-0d | 4.98 | <0.05 |
| OTU295 | Bacteroidetes | S24-7 | norank | Middle aged-0d | 4.22 | <0.05 |
| OTU418 | Firmicutes | Lactobacillaceae | Lactobacillus | Middle aged-0d | 3.96 | <0.05 |
| OTU346 | Firmicutes | Lactobacillaceae | Lactobacillus | Middle aged-0d | 4.36 | <0.05 |
| OTU368 | Firmicutes | Ruminococcaceae | uncultured | Middle aged-0d | 4.00 | <0.05 |
| OTU504 | Firmicutes | Lactobacillaceae | Lactobacillus | Middle aged-0d | 4.44 | <0.05 |
| OTU407 | Tenericutes | norank | norank | Middle aged-0d | 3.98 | <0.05 |
| OTU682 | Firmicutes | Peptostreptococcaceae | Incertae_Sedis | Middle aged-0d | 3.78 | <0.05 |
| OTU662 | Bacteroidetes | S24-7 | norank | Middle aged-0d | 4.00 | <0.05 |
| OTU286 | Firmicutes | Acidaminococcaceae | Phascolarctobacterium | Middle aged-14d | 4.81 | <0.001 |
| OTU769 | Firmicutes | Lachnospiraceae | Lachnospiraceae | Middle aged-14d | 3.97 | <0.05 |
| OTU200 | Bacteroidetes | Bacteroidaceae | Bacteroides | Middle aged-14d | 3.89 | <0.05 |
| OTU24 | Firmicutes | Ruminococcaceae | Ruminococcus | Middle aged-14d | 4.09 | <0.05 |
| OTU162 | Firmicutes | Ruminococcaceae | uncultured | Middle aged-14d | 4.46 | <0.05 |
| OTU765 | Bacteroidetes | Bacteroidaceae | Bacteroides | Middle aged-14d | 3.94 | <0.05 |
| OTU628 | Firmicutes | Peptococcaceae | uncultured | Middle aged-14d | 3.97 | <0.05 |
| OTU488 | Firmicutes | Ruminococcaceae | uncultured | Middle aged-14d | 4.34 | <0.05 |
| OTU779 | Bacteroidetes | Bacteroidaceae | Bacteroides | Middle aged-14d | 4.06 | <0.05 |
| OTU226 | Tenericutes | norank | norank | Middle aged-14d | 4.26 | <0.05 |
| OTU453 | Firmicutes | Defluviitaleaceae | uncultured | Middle aged-14d | 4.05 | <0.05 |
| OTU580 | Firmicutes | Ruminococcaceae | uncultured | Middle aged-14d | 4.08 | <0.05 |
| OTU661 | Tenericutes | norank | norank | Middle aged-14d | 4.13 | <0.05 |
